# AMP-activated protein kinase – not just an energy sensor

**DOI:** 10.12688/f1000research.11960.1

**Published:** 2017-09-22

**Authors:** David Grahame Hardie, Sheng-Cai Lin

**Affiliations:** 1 Division of Cell Signalling & Immunology, School of Life Sciences, University of Dundee, Dundee, UK; 2 State Key Laboratory of Cellular Stress Biology, Xiamen University, Xiang’an Campus, Xiamen, China

**Keywords:** AMP, activated protein kinase, ATP

## Abstract

Orthologues of AMP-activated protein kinase (AMPK) occur in essentially all eukaryotes as heterotrimeric complexes comprising catalytic α subunits and regulatory β and γ subunits. The canonical role of AMPK is as an energy sensor, monitoring levels of the nucleotides AMP, ADP, and ATP that bind competitively to the γ subunit. Once activated, AMPK acts to restore energy homeostasis by switching on alternate ATP-generating catabolic pathways while switching off ATP-consuming anabolic pathways. However, its ancestral role in unicellular eukaryotes may have been in sensing of glucose rather than energy. In this article, we discuss a few interesting recent developments in the AMPK field. Firstly, we review recent findings on the canonical pathway by which AMPK is regulated by adenine nucleotides. Secondly, AMPK is now known to be activated in mammalian cells by glucose starvation by a mechanism that occurs in the absence of changes in adenine nucleotides, involving the formation of complexes with Axin and LKB1 on the surface of the lysosome. Thirdly, in addition to containing the nucleotide-binding sites on the γ subunits, AMPK heterotrimers contain a site for binding of allosteric activators termed the allosteric drug and metabolite (ADaM) site. A large number of synthetic activators, some of which show promise as hypoglycaemic agents in pre-clinical studies, have now been shown to bind there. Fourthly, some kinase inhibitors paradoxically activate AMPK, including one (SU6656) that binds in the catalytic site. Finally, although downstream targets originally identified for AMPK were mainly concerned with metabolism, recently identified targets have roles in such diverse areas as mitochondrial fission, integrity of epithelial cell layers, and angiogenesis.

## Introduction

**Figure 1.**
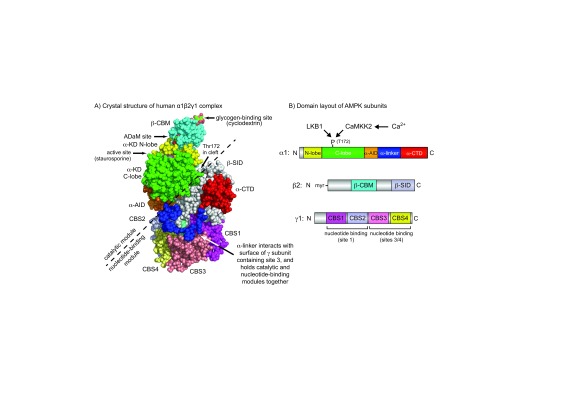
( **A**) Crystal structure based on Protein Data Bank file 4RER ^5^ and ( **B**) domain layout of the human α1β2γ1 complex of AMPK. The colour coding of domains in (A) and (B) is similar. Note that the catalytic module (above and left of the dashed line), comprising the α-KD, β-CBM, and α-AID, is a rather independent entity from the nucleotide-binding module (below and right of the dashed line), comprising the γ subunit, α-CTD, and β-SID. The α-linker is the principal “hinge” that connects these two modules. In this active conformation, the two modules are close together because the close interaction of the α-linker with AMP bound in site 3 pulls them together. However, when ATP rather than AMP occupies site 3, the α-linker is thought to dissociate from the γ subunit, allowing the two modules to move apart. This is thought to allow the α-AID to rotate back into its inhibitory position behind the α-KD ^5^ while also exposing phospho-Thr172 to protein phosphatases. In the active conformation in the picture, phospho-Thr172 is located around the back of the molecule in the cleft between the two modules. α-AID, alpha subunit auto-inhibitory domain; α-CTD, alpha C-terminal domain; α-KD, alpha subunit kinase domain; β-CBM, beta carbohydrate-binding module; β-SID, beta subunit interaction domain; AMPK, AMP-activated protein kinase; CBS, cystathionine-beta-synthase.

The AMP-activated protein kinase (AMPK) is best known as a sensor of cellular energy status in vertebrate cells ^1, 2^. The catalytic subunits (for which there are genes encoding alternate α1 and α2 isoforms in mammals) contain kinase domains (α-KDs) at their N-termini, and the small N-lobe and larger C-lobe (in yellow and green in Figure 1) are typical of serine/threonine kinase domains. The kinase domains are normally active only when phosphorylated at a threonine residue within the activation loop (usually termed Thr172 ^3^, although the exact numbering varies according to species and isoform). Thr172 phosphorylation is brought about by one of two principal upstream kinases: the tumour suppressor LKB1 or the Ca ^2+^/calmodulin-activated kinase CaMKK2 (CaMKK-β). The α-KD is followed by an auto-inhibitory domain (α-AID, in orange) that maintains the α-KD in an inactive conformation in the absence of AMP ^4, 5^. The α-AID is connected to the globular C-terminal domain (α-CTD, in red) by a region of more extended polypeptide termed the α-linker ^5– 8^ (in dark blue). The β subunits (β1 or β2) contain myristoylated N-terminal regions ^9^ (not present in the structure in Figure 1A), a central carbohydrate-binding module (β-CBM, in mid-blue) ^10, 11^ and a C-terminal subunit interaction domain (β-SID, in silver-grey). The latter forms the core of the complex by cross-linking the α-CTD to the N-terminal region of the γ subunit ^5, 6, 8, 12^. The γ subunits (γ1, γ2, or γ3) contain N-terminal regions of variable length, followed by four tandem repeats of sequence motifs known as cystathionine-beta-synthase (CBS) repeats that generate the binding sites for the regulatory nucleotides AMP, ADP, and ATP ^13^. The two pairs of repeats (CBS1:CBS2 and CBS3:CBS4) assemble head-to-head to form a disc-like structure with one repeat in each quadrant ^14^ (shown in various colours in Figure 1). This arrangement generates four potential ligand-binding clefts in the centre, although only three are used (site 1, between CBS1 and CBS2, and sites 3 and 4, between CBS3 and CBS4). Although all three may have to be occupied for maximal activation by AMP ^9^, the most critical appears to be site 3. Thus, mutations directly affecting AMP binding at this site (R531G ^13^ and R531Q ^15^) completely abolish activation by AMP, while in the active AMP-bound conformation the α-linker makes close contacts with residues that bind AMP in this site ^5– 8, 12^ ( Figure 1). Moreover, recent binding studies suggest that, as long as site 4 is occupied by AMP, site 3 binds AMP with higher affinity than ATP ^16^, again compatible with this being the critical regulatory site. Intriguingly, the α-linker is also the principal flexible “hinge” connecting two regions of the AMPK heterotrimer that are almost separate from each other: the “catalytic module” (containing the β-CBM, α-KD, and α-AID; top left in Figure 1A) and the “nucleotide-binding module” (containing the γ subunit, α-CTD, and β-SID; bottom right). Interactions between the α-linker and the γ subunit when AMP is bound at site 3 are thought to pull the two modules together, leading to the compact conformation shown in Figure 1A. Conversely, release of the α-linker from the γ subunit on displacement of AMP by ATP in site 3 is thought to allow the two modules to move apart, leading to a less compact conformation ^5, 17^.

## Canonical regulation of AMPK by energy stress

Stresses that interfere with catabolic production of ATP (for example, hypoxia, ischaemia, inhibition of glycolysis or mitochondrial ATP production) or that stimulate ATP consumption (for example, contraction of skeletal muscle) increase the cellular ADP:ATP ratio, analogous to the “battery” of the cell running flat. This is invariably accompanied by even larger increases in the AMP:ATP ratio, which are due to displacement of the adenylate kinase reaction (2ADP ↔ ATP + AMP) ^18^. Under these circumstances, AMPK is activated by three complementary mechanisms: (i) binding of AMP or ADP promotes Thr172 phosphorylation by LKB1 (possibly also by CaMKK2, although that is disputed ^19, 20^), (ii) binding of AMP or ADP inhibits Thr172 dephosphorylation by protein phosphatases ^6, 18, 19^, and (iii) binding of AMP but not ADP causes an allosteric activation of up to 10-fold ^18, 19^. The structural data suggest plausible mechanisms to explain mechanisms (ii) and (iii) since there is evidence that displacement of AMP by ATP at site 3 triggers dissociation of the α-linker from the γ subunit, causing the catalytic and nucleotide-binding modules to move apart ^5, 6^ (see previous section). This may not only allow the α-AID to rotate back into its inhibitory position behind the kinase domain ^4, 5^ but also expose Thr172, which in the compact conformation is partly buried in a deep cleft between the two modules ^6^ ( Figure 1A), thus allowing more rapid dephosphorylation. However, this model does not explain why it is only AMP (and not ADP) that causes allosteric activation, nor why AMP binding promotes Thr172 phosphorylation by LKB1.

Interestingly, AMPK complexes with different γ subunit isoforms (γ1, γ2, or γ3) display subtle variations in their responses to increases in AMP and ADP ^19^, suggesting that complexes at different locations could be tuned to respond differently to changes in adenine nucleotides, depending on which γ subunit isoform is present.

The LKB1 complex (comprising LKB1 and two accessory subunits, STRAD and MO25 ^21^) normally appears to be constitutively active ^22^, and the regulation of AMPK phosphorylation appears to be modulated instead by binding of adenine nucleotides to AMPK. However, AMPK activation by energy stress does not occur in many tumour cells that lack LKB1 (for example, HeLa cells) because the basal activity of CaMKK2 is too low to trigger significant Thr172 phosphorylation ^23^. The CaMKK2-AMPK pathway represents instead an alternate Ca ^2+^-activated pathway that mediates AMPK activation by hormones that release Ca ^2+^ from intracellular stores, such as thrombin ^24^ or ghrelin ^25^.

## Non-canonical activation by glucose starvation

When the yeast *Saccharomyces cerevisiae* is grown in high glucose, it uses fermentation (glycolysis to ethanol) almost exclusively to generate ATP. When glucose runs low, yeast needs to switch on (i) genes required for metabolism of other fermentable carbon sources such as sucrose or (ii) genes of mitochondrial oxidative metabolism, which are required for growth on low concentrations of glucose or on non-fermentable carbon sources such as ethanol. None of these metabolic adaptations occurs in the absence of genes encoding the α, β, or γ subunits of the AMPK orthologue, termed the SNF1 complex ^26^. The SNF1 complex is activated upon glucose starvation ^27, 28^ by phosphorylation of the threonine residue equivalent to Thr172 (Thr210) ^29^. Although glucose starvation is accompanied by large changes in the cellular ratios of AMP:ATP and ADP:ATP, the yeast SNF1 complex is not allosterically activated by AMP ^28^. Neither phosphorylation nor dephosphorylation of Thr210 appears to be sensitive to AMP either, although dephosphorylation may be inhibited by ADP ^30^. Thus, the ancestral role of the AMPK orthologue in unicellular fungi appears to have been in response to glucose starvation, but it remains unclear whether changes in adenine nucleotides are the crucial signals or whether there is instead some mechanism by which the SNF1 complex responds more directly to the availability of glucose.

It has been known for many years that glucose deprivation activates AMPK in mammalian cells ^31^, but it had generally been assumed that this effect was mediated by the canonical energy stress mechanism (that is, by increases in AMP:ATP or ADP:ATP ratios or both). However, recent studies suggest that the mammalian kinase may be able to sense glucose by a non-canonical mechanism independently of changes in adenine nucleotides. The first clue came with unexpected findings that Axin (a large adapter protein better known for its role in the Wnt signalling pathway) forms a ternary complex with LKB1 and AMPK in response to glucose starvation, thus bringing the upstream and downstream kinases together and promoting Thr172 phosphorylation ^32^. AMPK activation, both in mouse embryo fibroblasts (MEFs) starved of glucose *in vitro* and in livers of mice starved *in vivo*, was subsequently found to require not only Axin ^32^ but also Lamtor1 ^33^, a resident lysosomal protein that associates with the vacuolar ATPase (v-ATPase). Lamtor1 is a component of the pentameric Ragulator complex, which acts as a guanine nucleotide exchange factor (GEF) for RagA or RagB, whose GTP-bound forms trigger translocation of mTORC1 to the lysosome where it is activated ^34^. Axin, along with bound LKB1, was found to translocate to the surface of the lysosome upon glucose starvation, and these results suggested a model in which glucose deprivation led to the formation of a lysosomal complex involving v-ATPase, the Ragulator, Axin, LKB1, and AMPK, thus triggering AMPK activation ^33^. AMPK is known to phosphorylate Raptor (a key component of mTORC1) as well as the upstream regulator TSC2, thus causing rapid inhibition of mTORC1 ^35, 36^. However, glucose starvation still suppresses mTORC1 even in TSC2- and AMPK-null MEFs by causing dissociation of mTORC1 from the lysosome in a Rag GTPase-dependent manner ^37, 38^. Importantly, knockout of Axin in MEFs ** led to prolonged activation and much slower dissociation of mTORC1 from the lysosome after glucose starvation, most likely due to the ability of Axin to inhibit the GEF activity of the Ragulator complex ^33^. The ability of Axin to negatively regulate mTORC1 may also account for some of the beneficial roles of metformin ^39^. Taken together, these new findings suggest that the regulation of the AMPK and mTORC1 signalling pathways is much more closely intertwined than previously realised.

**Figure 2.**
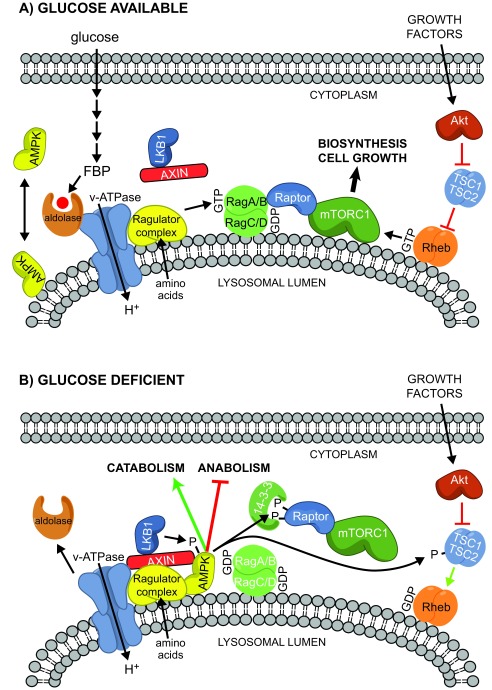
Working model for sensing of glucose availability by AMPK and its potential interactions with the mTORC1 signalling pathway at the lysosome. **(A)** When glucose is present, it is metabolised by glycolysis to FBP, which binds to aldolase and the v-ATPase at the lysosomal surface ^41– 43^, preventing the interaction of LKB1, AXIN, and AMPK on the lysosome. If amino acids are present (most likely within the lysosomal lumen ^78^), they activate the Ragulator complex, converting the RagA or RagB partner of the Rag heterodimer into its active GTP-bound form and recruiting mTORC1 to the lysosome because of the interaction between Raptor and RagA/B:GTP. If growth factors are also present, they activate the Akt pathway, inactivating the TSC1:TSC2 complex and promoting conversion of the small G protein Rheb into its active GTP-bound form. This further activates mTORC1, promoting biosynthesis and cell growth. Under these conditions, LKB1 is present as a complex with Axin in the cytoplasm, whereas AMPK may be partly cytoplasmic and partly lysosomal, and the latter location requires the N-terminal myristoylation of the β subunit. **(B)** When glucose is absent, FBP is no longer bound to aldolase and the latter may dissociate from the v-ATPase, at least in budding yeast. This allows a ternary complex of LKB1, AXIN, and AMPK to bind to the v-ATPase and the Ragulator complex, preventing activation of mTORC1. The proximity of LKB1 and AMPK also causes phosphorylation and activation of the latter. AMPK then phosphorylates Raptor (triggering 14-3-3 binding and preventing re-activation of mTORC1) and also TSC2, antagonising activation of mTORC1 by growth factors. AMPK also phosphorylates other targets that promote alternate catabolic pathways while inhibiting anabolic pathways. AMPK, AMP-activated protein kinase; FBP, fructose-1,6-bisphosphate; v-ATPase, vacuolar ATPase.

Although AMP can promote the formation of the complex between Axin and AMPK in reconstituted cell-free assays ^32^, it now appears that AMPK can sense glucose starvation independently of changes in adenine nucleotides ^40^. In MEFs, removing glucose from the medium (as long as glutamine and pyruvate were still present) caused rapid AMPK activation without any changes in AMP:ATP or ADP:ATP ratios. The AMPK activation that occurred upon glucose removal, but not the larger activation that occurred following energy stress (for example, on removal of both glucose and glutamine), was dependent on Axin, Lamtor1, and also N-terminal myristoylation of the AMPK-β subunits, and the last of these was required for lysosomal localisation of AMPK. The ability of glucose to repress AMPK activation required its metabolism by glycolysis as far as fructose-1,6-bisphosphate (FBP), and the sensor for glucose availability appears to be the glycolytic enzyme that metabolises FBP, i.e. aldolase ^40^. Intriguingly, aldolase has previously been shown to associate with the lysosomal v-ATPase in both yeast and mammalian cells ^41– 43^. These findings led to a model in which the lack of availability of glucose, and hence FBP, causes changes in the interaction between aldolase and the v-ATPase, promoting the formation of a complex between the Ragulator, Axin, LKB1, and AMPK ( Figure 2). Given that the association between aldolase and the v-ATPase is enhanced by glucose availability in yeast ^42^, it seems possible that elements of this mechanism are conserved between mammals and yeast and that glucose sensing by an AMP/ADP-independent mechanism is an evolutionarily ancient role of AMPK.

As well as these findings of binding of LKB1 to lysosomes, it has recently been reported that LKB1 can associate with specific plasma membrane compartments in cells from humans and *Drosophila melanogaster*. This appears to be due to basic regions in the C-terminal tail of LKB1 that cause its binding to phosphatidic acid and other phospholipids; mutation of these regions interferes with AMPK activation when LKB1 is expressed in HeLa cells ^44^.

## Role of ligands that bind the ADaM site

The β-CBM is a member of the CBM20 family of carbohydrate-binding modules, which are non-catalytic domains that usually occur in proteins that metabolise starch or glycogen. The β-CBM causes a proportion of mammalian AMPK to associate with glycogen particles in intact cells ^10, 11^, but the exact role of that remains unclear. However, another function of the β-CBM is that the cleft between it and the N-lobe of the α-KD forms a unique binding site accessible to various AMPK activators ^8, 12^. These compounds—including A-769662 ^45^, 991 ^12^, MT 63–78 ^46^, PF-06409577 ^47^, PF-739, PF-249 ^48^, and MK-8722 ^49^—all emerged from high-throughput screens that searched for allosteric activators of AMPK. They show varying selectivity for AMPK complexes containing the β1 rather than the β2 isoforms and are primarily allosteric activators, although they can also enhance net Thr172 phosphorylation by inhibiting dephosphorylation ^50, 51^. The extent of allosteric activation by A-769662 is particularly dramatic for AMPK that is not phosphorylated on Thr172. This effect requires prior autophosphorylation of Ser108 on the β subunit ^52^, although this is not required for activation by 991 ^53^. PF-739, which activates both β1- and β2-containing complexes, increased glucose uptake and lowered plasma glucose in diet-induced obese mice and healthy Cynomolgus monkeys, and in mice this was dependent on the expression of AMPK in muscle but not in liver. Thus, its effects appeared to be mediated by enhancing muscle glucose uptake rather than inhibiting hepatic glucose output ^48^. By contrast, the activator PF-249, which is β1-selective and therefore activates AMP in rodent liver but not in muscle, failed to lower plasma glucose or promote muscle glucose uptake, although PF-249 and another β1-selective activator, PF-06409577, showed promise in pre-clinical studies for treatment of diabetic nephropathy ^47^. Like PF-739, a similar “pan-β” activator, MK-8722, has shown promise in lowering blood glucose in both rodent and non-human primate models of type 2 diabetes ^49^. However, none of these compounds has yet progressed into clinical trials. The plant hormone salicylate also activates AMPK by binding at this site ^8, 54^. In the form of willow bark extract, salicylate has been used as a medicine since ancient times. It is also an *in vivo* breakdown product of aspirin (acetyl salicylate) and may exert some of the therapeutic effects of that drug. Salicylate is currently the only natural product known to bind this site on AMPK, but there is much speculation in the field that there may be a naturally occurring metabolite from animal cells that binds there, which is why it has been termed the allosteric drug and metabolite (ADaM) site ^55^.

## Paradoxical activation of AMPK by kinase inhibitors

It has recently been reported that AMPK is paradoxically activated by two kinase inhibitors: SU6656 ^56^ and sorafenib ^57, 58^. SU6656 was developed as an inhibitor of Src family kinases such as Src, Yes, and Fyn and was proposed to activate AMPK by inhibiting phosphorylation of tyrosine residues on LKB1 ^56^ or AMPK ^59^ by Fyn. However, activation of AMPK by SU6656 does not require phosphorylation of these tyrosine residues or even the presence of an Src family kinase in the cells ^60^. SU6656 is in fact a potent inhibitor of AMPK that binds at the catalytic site in competition with ATP, but this paradoxically causes a conformational change that promotes Thr172 phosphorylation by LKB1. This mechanism would still promote phosphorylation of downstream targets of AMPK as long as the lifetime of Thr172 phosphorylation was sufficient for SU6656 to dissociate and for one or more catalytic events to occur prior to Thr172 dephosphorylation. Indeed, SU6656 promotes the phosphorylation of the downstream target acetyl-CoA carboxylase in intact cells ^60^.

Sorafenib was originally developed as an inhibitor of tyrosine kinases and is used clinically for the treatment of hepatocellular carcinoma as well as advanced renal cell or thyroid carcinoma ^61^. Sorafenib also paradoxically activates AMPK ^57, 58^, but this is because sorafenib, in contrast to SU6656, is an inhibitor of the mitochondrial respiratory chain, so that it activates AMPK indirectly by increasing cellular AMP:ATP ratios ^60^.

## New targets: mitochondrial fission, maintaining the integrity of epithelial layers, and angiogenesis

**Figure 3.**
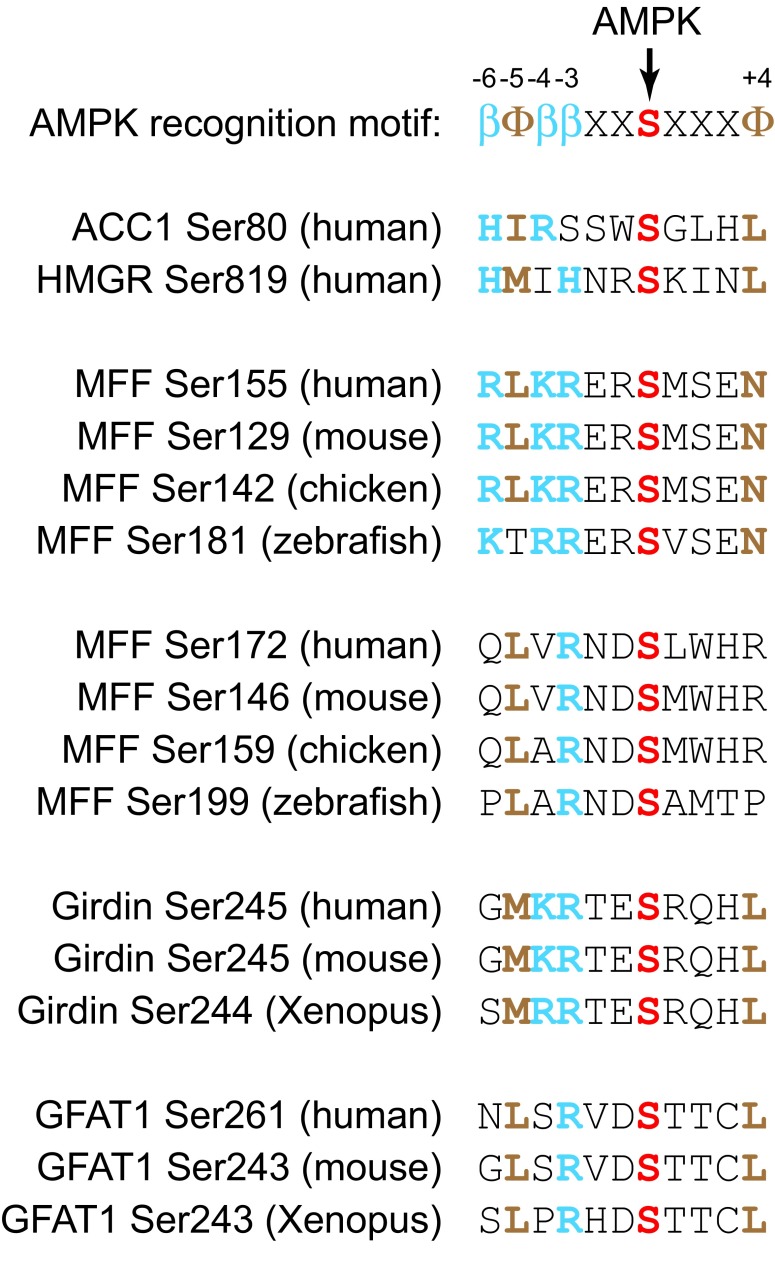
Alignment of consensus recognition motif for AMPK, classic sites phosphorylated by AMPK on acetyl-CoA carboxylase-1 (ACC1) and HMG-CoA reductase (HMGR), and novel sites recently identified on MFF, Girdin, and GFAT1. Basic residues at the -6, -4, and -3 positions are indicated in blue by the symbol “β” in the recognition motif and otherwise by the single-letter code in bold type. Hydrophobic residues at the -5 and +4 positions are indicated in brown by the symbol “Φ” in the recognition motif and otherwise by the single-letter code in bold type. Serine residues directly phosphorylated by AMPK are indicated in red bold type. In most cases, the sites were identified by using the human or mouse sequence, but the alignments show conservation of the sequences in other vertebrates. AMPK, AMP-activated protein kinase; GFAT1, glutamine:fructose-6-phosphate amidotransferase-1; MFF, mitochondrial fission factor.

AMPK phosphorylates serine or threonine residues within the recognition motif βΦ(X,β)XXS/TXXXΦ ^62^ ( Figure 3), where Φ represents bulky hydrophobic residues (M, L, I, F, or V) and β represents basic residues (R>K>H). The hydrophobic residue at the N-terminal (-5) position and at least one basic residue at either the -4 or the -3 position appear to be particularly critical. A recent review ^63^ listed over 60 well-validated targets of AMPK, and it is now clear that it phosphorylates many targets involved in cellular processes other than metabolism. A full review of these lies outside the scope of this article, but some recent interesting discoveries are mentioned below.

Mitochondria are now known to be extremely dynamic structures ^64^. Thus, respiratory chain inhibitors cause mitochondrial fission, possibly as a means of segregating regions of mitochondria that have undergone oxidative damage for subsequent autophagy and recycling. Mitochondria also become more fragmented in mitotic cells, perhaps to ensure even distribution to daughter cells. On the other hand, mitochondrial fusion into larger networks tends to occur in quiescent cells, which are more reliant on oxidative metabolism for ATP generation. Since mitochondria are the major suppliers of ATP in most quiescent cells and since AMPK knockout cells are known to accumulate abnormal mitochondria ^65– 67^, it is perhaps not surprising that AMPK should have a key role in mitochondrial dynamics. Indeed, in either U2OS cells or MEFs that lack both catalytic subunits of AMPK, the effects of mitochondrial inhibitors to trigger mitochondrial fission were attenuated ^68^. Moreover, compounds such as A-769662 (which activate AMPK by binding at the ADaM site) caused mitochondrial fission in the absence of energy stress. Fission is triggered by the GTPase dynamin-related protein-1 (DRP1), which is recruited to mitochondria in part by the mitochondrial fission factor (MFF). The latter is phosphorylated at two sites—Ser155 and Ser172—by AMPK ( Figure 3), and a phosphospecific antibody revealed that Ser172 was phosphorylated in intact cells treated with mitochondrial inhibitors or AMPK activators. Finally, localisation of DRP1 at mitochondria in response to mitochondrial inhibitors or AMPK activators was restored in MFF ^-/-^ cells reconstituted with wild-type MFF but not an S155A/S172A mutant ^68^. These results suggest that, as well as being involved in mitochondrial biogenesis ^69^ and mitophagy ^67^, AMPK may trigger mitochondrial fission. Thus, AMPK appears to be involved in the maintenance of mitochondrial function throughout the life cycle of these ATP-generating organelles.

The integrity and polarity of epithelial cell layers are other factors that are crucial to survival in vertebrates. Tight junctions between epithelial cells maintain a permeability barrier that helps to ensure that solutes and other materials such as pathogens cannot pass between cells but instead have to pass through them, where their uptake and onward transport can be monitored and regulated. Several years ago, it was shown that AMPK was activated during Ca ^2+^-induced tight-junction assembly in Madin-Darby canine kidney (MDCK) epithelial cells and that AMPK activators also protected tight junctions from disassembly induced by Ca ^2+^ depletion ^70, 71^. However, the direct target(s) of AMPK responsible for these effects remained unclear. One candidate to explain these effects is Gα-interacting vesicle-associated protein (GIV), also known as Girdin. AMPK phosphorylates Ser245, a good fit to the AMPK consensus motif that is located in the junction between the N-terminal and coiled-coil domains of Girdin ( Figure 3). This was observed both in cell-free assays and in MEFs subjected to glucose starvation, and the signal in the latter case disappeared in AMPK knockout cells ^72^. In MDCK cells, AMPK and Girdin phosphorylated at Ser245 did not co-localise with tight junctions in cells that were fully polarised under basal conditions but did so in cells subject to stress (glucose starvation or Ca ^2+^ depletion), when tight junctions are known to be turning over. Evidence was obtained by expressing non-phosphorylatable (S245A) and phosphomimetic (S245D) mutants in type II MDCK cells (which have low Girdin expression), suggesting that Ser245 phosphorylation was responsible for maintaining tight-junction integrity during glucose starvation. Ser245 phosphorylation also appeared to be responsible for interaction of Girdin with microtubules that are associated with tight junctions.

A recent phosphoproteomic screen in wild-type and AMPK-null MEFs treated with the ADaM site ligand A-769662 identified Ser-243 on glutamine:fructose-6-phosphate amidotransferase-1 (GFAT1) as an AMPK target ^73^ ( Figure 3). GFAT1 catalyses the formation of glucosamine-6-phosphate, the first and possibly rate-limiting step in the pathway of formation of UDP- *N*-acetylglucosamine, which is used to modify serine/threonine residues on numerous proteins with N-acetylglucosamine. Although Ser-243 had been suggested previously to be an AMPK site ^74– 76^, the effects on GFAT activity were unclear. In the new study, evidence was obtained that AMPK is a negative regulator of this pathway. Thus, AMPK inhibits the synthesis of N-acetylglucosamine as well as many other biosynthetic pathways. In endothelial cells, the hexosamine biosynthesis pathway, which is enhanced by high glucose availability, is a negative regulator of angiogenesis, while phosphorylation of GFAT1 by AMPK (for example, after activation by vascular endothelial growth factor mediated by the CaMKK2 pathway ^77^) promotes angiogenesis ^73^. Thus, AMPK appears to be critical in enhancing angiogenesis, a process that would be beneficial in nutrient-deprived cells.

## Conclusions and perspectives

Although important questions remain, good progress has been made in obtaining structural data that provide insights into the molecular mechanisms by which the AMPK heterotrimer is activated by the canonical energy-sensing mechanism involving changes in AMP, ADP, and ATP. At the same time, it has become clear that AMPK is activated by glucose starvation by a lysosomal mechanism that is independent of changes in adenine nucleotides; this may even have evolved before the energy-sensing mechanism and may represent the ancestral role of the AMPK system. The number of pharmacological agents that activate AMPK has continued to increase, including several that bind in the so-called “ADaM” site and at least one (SU6656) that binds in the catalytic site yet causes paradoxical activation. Finally, the number of well-validated downstream targets for AMPK has continued to increase, including targets involved in mitochondrial fission, in the maintenance of tight junctions, and in the N-acetylglucosamine synthesis pathway.

## Abbreviations

α-AID, alpha subunit auto-inhibitory domain; α-CTD, alpha C-terminal domain; α-KD, alpha subunit kinase domain; β-CBM, beta carbohydrate-binding module; β-SID, beta subunit interaction domain; ADaM, allosteric drug and metabolite; AMPK, AMP-activated protein kinase; CBS, cystathionine-beta-synthase; DRP1, dynamin-related protein-1; FBP, fructose-1,6-bisphosphate; GEF, guanine nucleotide exchange factor; MDCK, Madin-Darby canine kidney; MEF, mouse embryo fibroblast; MFF, mitochondrial fission factor; v-ATPase, vacuolar ATPase.
